# Intestinal Gel-Forming Mucins Polymerize by Disulfide-Mediated Dimerization of D3 Domains

**DOI:** 10.1016/j.jmb.2019.07.018

**Published:** 2019-09-06

**Authors:** Gabriel Javitt, María Luisa Gómez Calvo, Lis Albert, Nava Reznik, Tal Ilani, Ron Diskin, Deborah Fass

**Affiliations:** Department of Structural Biology, Weizmann Institute of Science, Rehovot 7610001, Israel

**Keywords:** D1D2D3, mucin 2 recombinant protein spanning residues 21 to 1259, D1D2D3CysD1, mucin 2 recombinant protein spanning residues 21 to 1397, DTT, dithiothreitol, endoH, endoglycosidase H, MUC2, mucin 2, MUC2D3, mucin 2 recombinant protein spanning residues 858 to 1259, SDS-PAGE, sodium dodecyl sulfate polyacrylamide gel electrophoresis, SEC-MALS, size exclusion chromatography with multi-angle light scattering, VWF, von Willebrand factor, mucin, disulfide bonds, quaternary structure, Golgi, colon

## Abstract

The mucin 2 glycoprotein assembles into a complex hydrogel that protects intestinal epithelia and houses the gut microbiome. A major step in mucin 2 assembly is further multimerization of preformed mucin dimers, thought to produce a honeycomb-like arrangement upon hydrogel expansion. Important open questions are how multiple mucin 2 dimers become covalently linked to one another and how mucin 2 multimerization compares with analogous processes in related polymers such as respiratory tract mucins and the hemostasis protein von Willebrand factor. Here we report the x-ray crystal structure of the mucin 2 multimerization module, found to form a dimer linked by two intersubunit disulfide bonds. The dimer structure calls into question the current model for intestinal mucin assembly, which proposes disulfide-mediated trimerization of the same module. Key residues making interactions across the dimer interface are highly conserved in intestinal mucin orthologs, supporting the physiological relevance of the observed quaternary structure. With knowledge of the interface residues, it can be demonstrated that many of these amino acids are also present in other mucins and in von Willebrand factor, further indicating that the stable dimer arrangement reported herein is likely to be shared across this functionally broad protein family. The mucin 2 module structure thus reveals the manner by which both mucins and von Willebrand factor polymerize, drawing deep structural parallels between macromolecular assemblies critical to mucosal epithelia and the vasculature.

## Highlights

• Network formation by gel-forming mucins protects epithelia but is poorly understood
• Intestinal mucin N-terminal module dimerizes rather than trimerizes
• The mucin dimer forms through atypical protein-protein interaction sites
• Gut mucins and a blood hemostasis factor share polymerization mechanisms

## Graphical abstract

**Figure:**
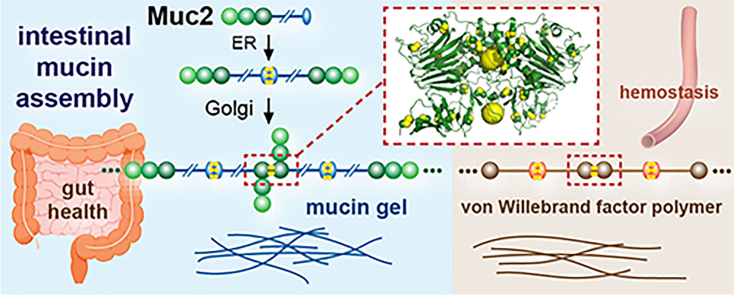

## Introduction

Mucin glycoproteins are the main structural component of mucus and thus play a central role in protecting exposed epithelia from pathogens and mechanical damage [1], [2]. The distinctive amino acid composition, extensive glycosylation, and higher-order assembly of mucins determine their physical and biological properties. In particular, multimerization of mucins is key to their activity. The viscosity and porosity of mucus are likely to be affected by the density of junctions in the mucin network [3]. The extent of cross-linking during biosynthesis followed by the proteolytic degradation of secreted mucin oligomers may affect the observed turnover of mucins [4] and together determine the rate at which mucus and its trapped contents are flushed from luminal cavities of the body.

**Fig. 1:**
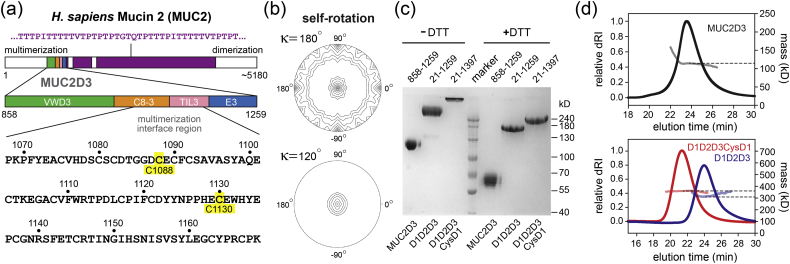
MUC2D3 organization. (a) Primary structural map of human MUC2 (Uniprot Q02817) shows the location of MUC2D3 within the full-length protein, the domain composition of MUC2D3, and the location of the intersubunit disulfide bonds (highlighted in yellow) in the amino acid sequence. A representative segment of the low-complexity (threonine-rich) sequence (purple) is displayed. (b) The κ = 180° (top) and κ = 120° (bottom) sections of the self-rotation function of the MUC2D3 diffraction data show that molecules in the crystal are related by a rotation of ~ 120° around an axis coincident with a crystallographic 2-fold symmetry axis. (c) SDS-PAGE (7.5% gel) of glycosylated MUC2D3 and longer MUC2 fragments without reduction by dithiothreitol (− DTT) and following reduction (+ DTT). Amino acid ranges for each fragment are given above the gel, and the corresponding domain designations are shown below. D1D2D3 spans MUC2 residues 21 to 1259; D1D2D3CysD1 spans residues 21 to 1397. (d) SEC-MALS analysis of MUC2D3 (above) and longer MUC2 fragments (below). The signals from the differential refractrometer (dRI) were normalized to yield peak values of 1 for MUC2D3 and D1D2D3CysD1, and the dRI signal of D1D2D3 was scaled by the same factor as D1D2D3CysD1. Dashed lines intersect the plots of molecular mass at the dRI peaks. The higher masses detected at the leading edge of the MUC2D3 peak are likely to be due to some non-covalent aggregation of dimers, rather than to a higher-order covalent assembly, because no hint of additional covalent multimers was seen by SDS-PAGE, even in overloaded gels. Calculated subunit masses (protein component only and including the carboxy-terminal His_6_ tag) are as follows: 45,700 Da for MUC2D3; 137,500 for D1D2D3, and 152,700 for D1D2D3CysD1.

Mucin multimerization occurs in a series of steps in the secretory pathway. The mucin protomer contains amino- and carboxy-terminal oligomerizing regions separated by a central serine- and threonine-rich segment heavily modified by O-linked glycans (Fig. 1a). In the first step of quaternary structure assembly, mucins dimerize by covalent association of their carboxy-terminal regions [5]. Dimerization was shown to occur prior to glycan processing in the Golgi and to be insensitive to brefeldin A treatment, suggesting that this first assembly step occurs in the endoplasmic reticulum (ER) [5]. The second major event in mucin multimerization occurs by covalent association of the amino-terminal regions. Based on the inhibition of amino-terminal fragment multimerization upon treatment of cells with various agents that indirectly affect Golgi function, it was concluded that this second assembly step occurs in the Golgi apparatus [6].

Certain aspects of domain organization and assembly mechanism are shared among intestinal mucin 2 (MUC2), other mucins such as those in the respiratory tract (MUC5b and MUC5ac), and von Willebrand factor (VWF), a protein involved in blood homeostasis. Each of these proteins contains the carboxyl-terminal dimerization region and the amino-terminal multimerization domains. The carboxy terminal regions appear to function similarly in the homologous proteins, forming dimers through tightly interacting β-ribbons affixed by three intermolecular disulfide bonds, as represented by the VWF cystine knot domain [7]. Although the mucin and VWF dimers linked by this motif are all proposed to further polymerize *via* amino-terminal domains, the oligomerization states of these domains have been suggested to differ. Specifically, recombinant intestinal and submaxillary mucins are proposed to trimerize through their amino-terminal regions [6], [8], [9], whereas lung mucins and VWF are proposed to dimerize using homologous domains [10], [11]. These differences are expected to produce polymers with different properties that are adapted to their particular local conditions and functions [12]. For example, linear polymers of VWF efficiently coat sites of vasculature wounding under shear stress, whereas intestinal mucin networks are appropriate for protecting the large exposed interior surface of the gut. A current model for colon mucin assembly suggests that disulfide-mediated trimerization of carboxy-terminally linked MUC2 dimers produces a hexagonal lattice, which contributes to forming the laminated structure coating the epithelium, when the mucins are secreted by the goblet cells of the gut [9], [13]. The dimensions of this putative lattice would be determined in part by the lengths of the threonine-rich region between the amino- and carboxy-terminal globular domains [14]. A mesh with pore sizes of hundreds of nanometers would effectively filter out microorganisms from the epithelial surface but readily allow diffusion of ions, metabolites, and proteins [15].

Despite the dominance of this model in the literature, the possibility of alternate multimerization modes for intestinal mucins has been raised [16]. Furthermore, the structural and mechanistic basis for the proposed differences in quaternary assembly of members of the mucin/VWF protein family has remained unclear. VWF migrates as a ladder when separated by electrophoresis in gels under non-reducing conditions, representing variation in the number of covalently-bound subunits in the polymer [17]. Determining the oligomerization state of intact, non-reduced MUC2 assemblies is not straightforward, as human MUC2 contains over 5000 amino acids, may bear four times the mass of the polypeptide in carbohydrates [18], and is cross-linked by isopeptide bonds as well as disulfides [19]. Thus, a direct comparison between VWF and MUC2 is complicated. However, structural analysis of recombinant fragments can provide insights that are likely relevant to full-length mucin and VWF proteins (e.g., Ref. [7]). To explore the mechanism of disulfide-linked multimerization of the MUC2 amino-terminal region, we determined the crystal structure of a recombinant MUC2 fragment containing the VWD3 domain and investigated the oligomerization state of the entire amino-terminal segment spanning almost 1400 amino acids. We show that MUC2 forms amino-terminal dimers instead of the anticipated trimers. This finding has wide-ranging implications for the multimerization mechanism of intestinal mucins and other distinct but homologous biological assemblies.

## Results

### Production and crystallization of the mucin 2 multimerization module

The human MUC2 region spanning the VWD3, C8–3, TIL-3, and E-3 domains (Fig. 1a) [20] was produced in human embryonic kidney HEK293F cells and purified from the medium. Small crystals were obtained, but they diffracted poorly. The crystallized MUC2 fragment contains six potential N-linked glycosylation sites, one in the VWD3 domain and the rest in the TIL-3 or E-3 domains (Supplemental Fig. 1). To enable removal of flexible glycans, the same MUC2 fragment (hereafter referred to as MUC2D3) was produced in cells grown in the presence of kifunensine, an inhibitor of high-mannose carbohydrate processing [21]. MUC2D3 containing high-mannose sugars was purified and treated with endoH to leave only a monosaccharide at glycosylated asparagines. The endoH-treated protein produced larger and better-ordered crystals, in an orthorhombic space group. Diffraction data were collected to 2.7 Å resolution (Supplemental Table 1).

A self-rotation function performed on the MUC2D3 diffraction data set produced the expected peaks for the orthogonal 2-fold rotation axes corresponding to the crystallographic symmetry and an additional peak consistent with a rotation of 120° (Fig. 1b). The 120° rotation would not have been surprising, as MUC2D3 was expected to be a trimer [6], [8], [9]. However, on denaturing polyacrylamide gels the protein purified from cell culture supernatants migrated at the apparent molecular mass expected for a dimer (Fig. 1c), and size exclusion chromatography with multi-angle light scattering (SEC-MALS) showed a solution molecular mass of a dimer (Fig. 1d). For comparison, two longer fragments spanning all the VWD domains in the amino-terminal region of MUC2 were expressed and purified. The non-reduced versions of these proteins migrated in gels above the highest molecular mass marker (Fig. 1c), but SEC-MALS showed masses for the folded complexes in solution consistent with dimers. Even the largest fragment, which contained the CysD1 domain following a short threonine-rich segment (Supplemental Fig. 2) and migrated very slowly in gels under non-reducing conditions (Fig. 1c), formed a dimer according to SEC-MALS (Fig. 1d). To determine whether the observed oligomerization state was specific to HEK293F cells, we transfected the MUC2D3 plasmid and the plasmid containing the entire amino-terminal region of MUC2 into cells of the Caco-2 line (epithelial colon-derived adenocarcinoma). The same electrophoretic gel migration pattern was seen for the proteins produced in Caco-2 cells (Supplemental Fig. 3).

### Structure of the mucin 2 multimerization module resolved the oligomerization state

**Fig. 2:**
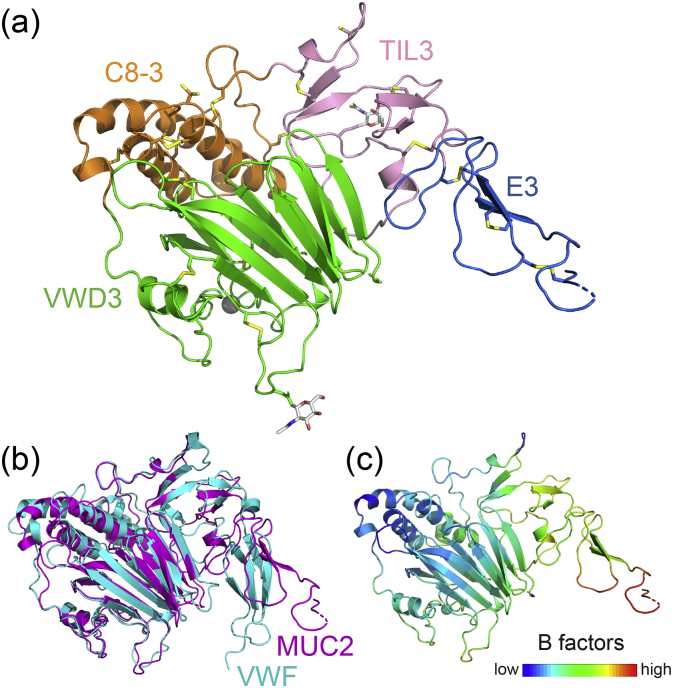
MUC2D3 protomer structure. (a) A ribbon diagram illustrating the structure of MUC2D3 (chain A) is colored and oriented for comparison with VWF (22). Disulfide bonds and glycans are shown in stick format. A bound calcium ion is represented as a gray sphere. (b) Superposition of MUC2D3 (magenta) with the homologous region of VWF (teal). The two structures have a Cα RMSD of 2.8 Å and a TM score of 0.84 over 333 amino acids according to TM-align [23]. (c) The MUC2D3 structure colored according to temperature factor (*B* factors) shows that the E-3 and TIL-3 domains are more flexible than the VWD3 and C8–3 domains.

Phases for determining the MUC2D3 structure were obtained by molecular replacement using the monomeric VWF D3 domain (PDB ID: 6N29) [22]. The search model consisted of VWF residues 865 to 1227, which includes the VWD3, C8–3, and TIL-3 domains, and part of the E-3 domain. Four MUC2D3 molecules were identified in the asymmetric unit. The MUC2D3 structure was iteratively rebuilt and refined against the diffraction data. The most complete molecule in the asymmetric unit was chain A (Fig. 2a). The structure of the MUC2D3 protomer shares the constituent folds and overall organization of the homologous regions of VWF (Fig. 2b), with the exception that E-3 is not packed as tightly against the VWD3 β-sandwich as in VWF. This fact may have contributed to the relative disorder of E-3 in the MUC2D3 crystals. The temperature factors are lowest in the C8–3 region and highest in the E-3 domain (Fig. 2c).

**Fig. 3:**
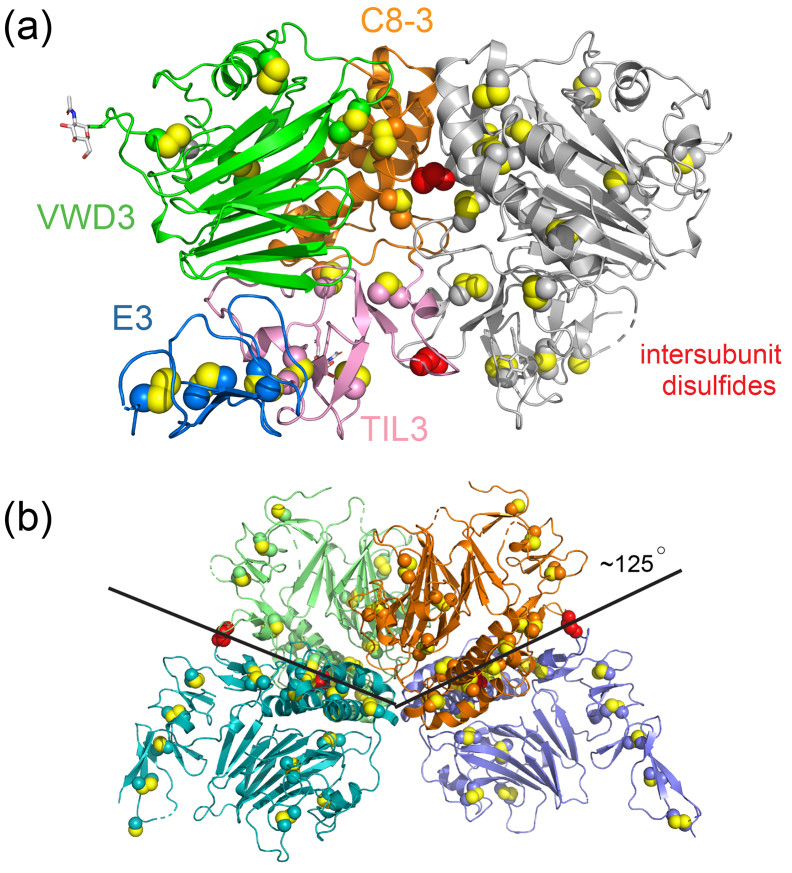
MUC2D3 multimerization. (a) MUC2D3 dimer with one subunit colored as in Fig. 2a and another in gray. Atoms in cysteine side chains are shown as spheres, with the intersubunit disulfides colored red. (b) The two MUC2D3 dimers in the asymmetric unit are shown with the intersubunit disulfides colored red. The packing angle between the dimers, which produced the peak on the κ = 120° section of the self-rotation function, is indicated.

The proposed model for MUC2 assembly involves formation of disulfide bonded dimers linked by a carboxy-terminal domain, followed by trimerization *via* the VWD3 region [6], [8], [9]. However, the MUC2D3 protomers in the crystal asymmetric unit were found to form two disulfide-linked dimers rather than a trimer (Fig. 3a), consistent with the sodium dodecyl sulfate polyacrylamide gel electrophoresis (SDS-PAGE) and SEC-MALS analyses of the MUC2 fragments (Fig. 2c, d). Examination of the packing of MUC2D3 in the crystals revealed that the two disulfide-linked dimers were related by a rotation of about 125° around an axis parallel to the crystal c axis (Fig. 3b), explaining the peak on the 120° kappa section in the self-rotation function (Fig. 1b). No actual trimer was detected in the subunit arrangement in the crystal. Crystallographic symmetry produced a complex of four dimers arranged with the VWD3 and C8–3 domains toward the center and the TIL-3 and E-3 domains on the surface (Supplemental Fig. 4).

In addition to revealing the multimerization mode of the MUC2 VWD3 region, the crystal structure illuminated the pattern of intermolecular disulfide bonding and the nature of the intersubunit interface. Cys1130, located in the TIL-3 domain, was suggested to be involved in MUC2 oligomerization [6], and its participation was confirmed by the MUC2D3 structure, in which Cys1130 bonds with its symmetry-mate across the dimer interface. It was previously assumed that a second cysteine would be involved, as required for trimerization [6]. Although a second cysteine is not necessary for dimerization, Cys1088, located at the amino terminus of a helix in the C8–3 domain, was found to form an additional intersubunit disulfide. These two disulfides are about 27 Å apart from one another in the interface. With the exception of the abutting amino termini of the Cys1088 helices, the interface is devoid of regular secondary structure (Fig. 3a). The buried solvent-accessible surface area is about 1230 Å^2^, a relatively low value even compared with a set of non-obligate multimers [24].

**Fig. 4:**
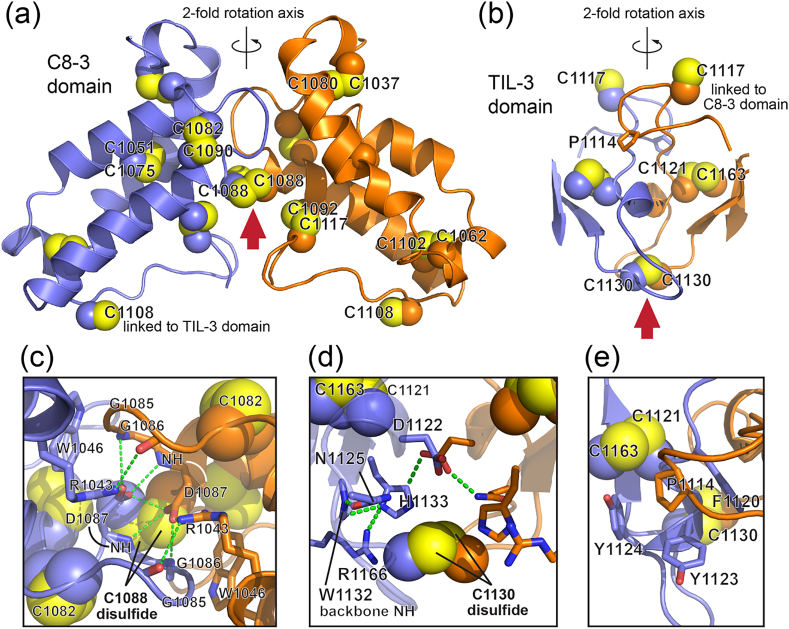
Environments of the disulfides in the MUC2 multimerization interface. (a) The two C8–3 domains (violet and orange) in the MUC2D3 dimer are related by a 2-fold axis of rotation around the vertical. Atoms in cysteine side chains are shown as spheres. The red arrow indicates the C1088–C1088 intersubunit disulfide from the solvent-exposed direction. (b) The two TIL-3 domains are presented with the 2-fold rotation axis vertical. The red arrow indicates the C1130–C1130 intersubunit disulfide. (c) Some of the key polar side chains in the interface above the C1088–C1088 disulfide are shown as sticks and labeled. Green dashed lines are putative hydrogen bonds. (d) Some of the key polar side chains in the interface above C1130–C1130 are shown as sticks and labeled. Green dashed lines are putative hydrogen bonds. A duplicate set of symmetry-related interactions also exist but for clarity are not shown. (e) A hydrophobic interaction is made by the packing of a conserved proline into a pocket formed by the indicated aromatic side chains in the second subunit. The reciprocal interactions also occur but are not seen in this view.

The two intersubunit disulfides involve cysteines in distinct domains (Fig. 4a, b). C1088 is adjacent to a hydrogen bond network formed between charged side chains (R1043 and D1087) and the polypeptide backbone of the opposite subunit (Fig. 4c). Interestingly, the C1088–C1088 disulfide is asymmetric; the two cysteines occupy different rotamers (Supplemental Fig. 5). Another set of intersubunit polar interactions is found in TIL-3 near C1130, involving D1122, N1125, H1133, and R1166 (Fig. 4d). Each of the intersubunit disulfides is partially solvent-exposed (red arrows in Fig. 4a, b). In addition to the areas around the two intermolecular disulfides, a third point of contact between the subunits in the dimer is observed midway between the disulfides, where a proline side chain (P1114) (Fig. 4b) inserts into a hydrophobic pocket lined on three sides by aromatic side chains (F1120, Y1123, Y1124) and on the fourth by an intrasubunit disulfide (C1121–C1163) (Fig. 4e). The small and predominantly polar multimerization interface observed in MUC2D3 may explain why it does not form readily in the early stages of protein assembly and why it may require a particular pH range, ion concentration, or other factors found further along the secretory pathway.

**Fig. 5:**
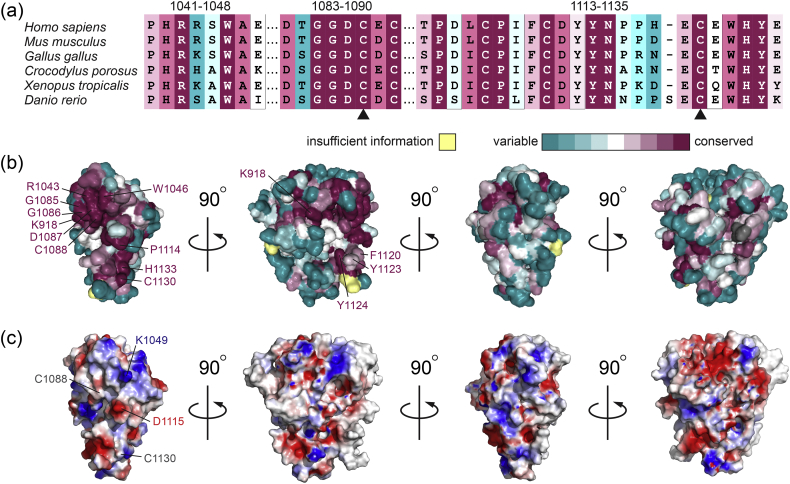
Conservation of surface residues in MUC2 orthologs. (a) Color-coded MUC2 sequence alignment of representative organisms, extracted from a Consurf analysis [25] of a larger number of orthologs. Selected segments of the alignment are shown, covering most of the conserved, labeled surface residues in panel b. (b) Colors of the Consurf analysis mapped onto the surface of the MUC2D3 protomer. (c) Electrostatic potential generated using Pymol mapped to the MUC2D3 surface.

To assess the likelihood that the observed dimer interface and intersubunit cysteine connectivity reflect an evolved interaction in MUC2, we analyzed the amino acid conservation of the multimerization domain across MUC2 orthologs (Fig. 5a) and displayed conservation scores on the domain surface using Consurf [25] (Fig. 5b). The regions involved in dimerization are the most highly conserved on the surface of the protein, with important residues being invariant. For example, G1085, G1086, and D1087 are completely conserved, as are R1043 and W1046 (Fig. 4c). In the region of C1130, H1133 (Fig. 4d) is invariant. Even when minor variation is observed, as in F1120 and Y1123 of the hydrophobic pocket that binds the invariant P1114, these residues are seen to be replaced only by other aromatics, which could perform the same structural role. Conversely, positions observed to be more variable, such as D1115, are solvent-exposed despite their proximity to the dimer interface. The striking conservation of the dimer interface residues contrasts with the apparent lack of uniqueness of the surface electrostatics of this face of the protein (Fig. 5c). Notably, the cysteines involved in dimerization are missing in the D1 and D2 domains of MUC2 (Supplemental Fig. 6), as expected since these domains do not make intermolecular disulfide bonds.

### Implications for von Willebrand factor and other mucins

**Fig. 6:**
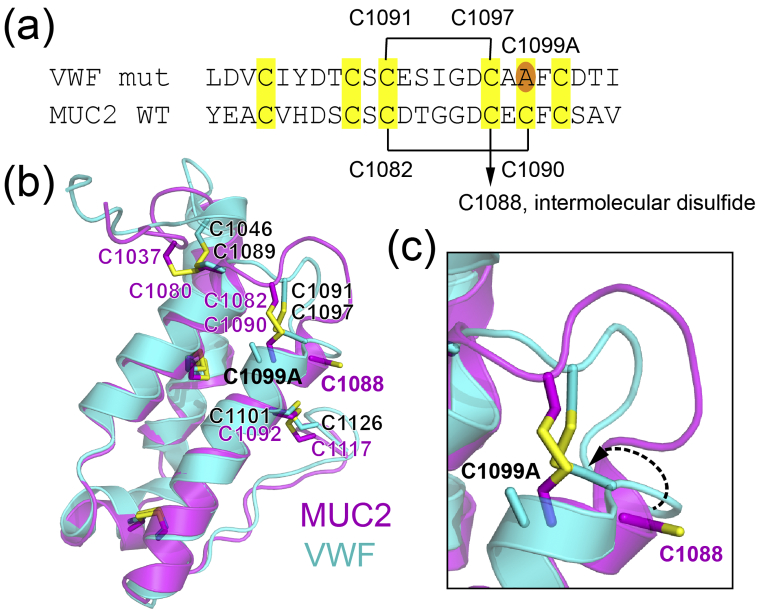
Comparison of MUC2 and VWF cysteines participating in the first intermolecular disulfide. (a) Amino acid sequence alignment between MUC2 and VWF in the region surrounding MUC2 C1088. Disulfide connectivity is indicated by black lines connecting cysteines. (b) The MUC2 and VWF structures were superposed over amino acids 1067 to 1118 (MUC2) and 1076 to 1027 (VWF). MUC2 is magenta; VWF is cyan. The MUC2 interface cysteine C1088 and the mutated VWF position C1099A are shown in bold. (c) The local conformational change that may have resulted in an alternate cysteine pairing in the VWF mutant is suggested by a curved, dashed arrow.

A comparison of the MUC2D3 structure with VWF sheds light on the assembly mechanism of both proteins. VWF and MUC2 share 38% sequence identity over the VWD3, C8–3, and TIL-3 domains (Supplemental Fig. 7). The cysteine residues are conserved throughout VWD3 and most of C8–3. The MUC2D3 structure presented here contained its full complement of cysteines, but the VWF structure was determined using a mutant designed to be monomeric [22]. To generate the monomeric version, the VWF amino acid comparable to MUC2 C1130 (VWF C1142) was mutated to alanine (Fig. 6a). In addition, the VWF amino acid comparable to MUC2 C1090 (VWF C1099) was mutated. However, in the MUC2 structure, C1090 forms an intramolecular disulfide with C1082, and C1088 is instead the intermolecular disulfide as described above. There are two possible explanations for this discrepancy. Either VWF and MUC2 use different cysteines for dimerization or the true interface cysteine in VWF is C1097, which is the cysteine aligned with the MUC2 interface cysteine C1088 (Fig. 6a). According to the latter explanation, VWF C1097 would have flipped inwards to replace the mutated cysteine VWF C1099 and form a non-native partnership in the mutant VWF structure (Fig. 6c). This issue is further discussed below.

Despite their overall similarity and the conservation of the cysteines in the region of the intersubunit disulfides, MUC2 and VWF do show some differences. First, the MUC2 proline pocket displayed in Fig. 4e is absent in VWF: the proline is replaced by alanine, and the three aromatic residues are instead serine, glutamate, and arginine (Supplemental Fig. 7). In addition, C1137 in MUC2 and its counterpart in VWF, C1149, though aligned in sequence and thus apparently conserved, have different intramolecular disulfide bonding partners. MUC2 C1137 bonds to a cysteine upstream in the sequence, in the C8–3 region, whereas VWF C1149 bonds to a downstream cysteine (Supplemental Fig. 7). In the three-dimensional structure, the conserved cysteines are found roughly in the same position, but the side chains face different directions to accommodate their different partners. VWF also has an additional disulfide in the TIL-3 domain that is absent in MUC2 (Supplemental Fig. 7). Overall, however, the MUC2 and VWF D3 and associated domains are remarkably similar considering the different physiological functions of these two proteins and their evolutionary distance.

Given the similarity of MUC2 and VWF, it is not surprising that the regions important for MUC2 multimerization are also generally observed in other gel-forming mucins. Both C1088 and C1130, along with other interface residues (Supplemental Fig. 8), are conserved in human MUC5ac, MUC5b, and MUC6. MUC19, however, is missing the interface cysteine comparable to MUC2 C1088, as well as the cysteine comparable to MUC2 C1090. Other highly conserved residues in the vicinity of the missing cysteines are also altered in MUC19, so it is likely that this region is structurally divergent in that mucin.

## Discussion

The epithelial cells of organs including the lung and intestines cover enormous surface areas of the body that, though located internally, are exposed to biological and physical threats originating in the outside world. These sensitive surfaces are protected due to the multimerization properties of gel-forming mucins such as MUC2. The size and complexity of mucins and, even more-so, of mucin multimers, makes the investigation of their self-assembly mechanism very challenging. Successive intermolecular disulfide bonding steps result in the formation of supramolecular mucin networks, but the structures of the domains involved in some of these steps and the manner in which they are linked have been elusive.

In this study, we present the oligomeric state and covalent interaction interface of the human mucin 2 multimerization domain. In contrast to the predominant model in the field, the MUC2 amino-terminal region was found to form disulfide-linked dimers rather than trimers. No other oligomerization state was detected in preparations of MUC2 amino-terminal fragments expressed recombinantly from human cells (Fig. 1c). Despite some conformational heterogeneity in the MUC2D3 crystal, particularly in the E-3 region, the dimer interface geometry and the disulfide connectivity throughout the VWD3, C8–3, and TIL-3 domains were homogeneous and well resolved. The only exception was that a minor fraction of the C1088–C1088 intermolecular disulfides may have become reduced in the crystals upon intense x-ray irradiation, a phenomenon that has been observed for other proteins [26], [27]. In physiological contexts, the need to reduce two different disulfides in two domains to disassemble the MUC2 amino-terminal dimer may contribute to the stability of this assembly.

Mucin paralogs in some organs are considered to dimerize *via* amino-terminal regions [16], [28], whereas other paralogs were proposed to trimerize through homologous regions [6], [8], [9]. While not impossible, this model is less parsimonious than one in which homologous domains assume the same oligomerization state. A number of arguments support the physiological relevance of the dimeric oligomerization state of MUC2D3. First, polar dimer interface amino acids and amino acids involved in a key intermolecular hydrophobic interaction are highly conserved, with many of these residues remaining invariant in MUC2 orthologs across chordates. Second, protein quaternary structures tend to evolve by accumulating additional symmetry elements rather than by exchanging symmetries [29]. For example, paralogous proteins may be found as dimers or tetramers, the latter formed by evolution of a new interface to create a dimer of dimers. In contrast, it is rare for homologous proteins to exhibit quaternary structures that require breaking and reforming interfaces, as necessary for forming trimers *versus* dimers. Dimerization through disulfide bonds may involve reciprocal interactions between one or more cysteines of the two protomers, while trimerization requires participation of at least two cysteine residues per protomer linked in a non-reciprocal manner (as in Protein Data Bank IDs: 2YF2, 4B0F, and 3DUZ). Thus, a rearrangement of non-reciprocal to reciprocal disulfide bonds would represent a major change in the multimerization interface.

If the MUC2 VWD3 region indeed forms covalent dimers rather than trimers, one must reconsider the evidence that suggested trimers. A standard method for determining protein sizes is gel electrophoresis, but assembled amino-terminal fragments of MUC2 run above the highest molecular mass marker in commercial ladders (Fig. 1c). Importantly, migration of proteins on gels will be affected not only by absolute molecular mass but also by cross-linking, branching, glycosylation, and SDS binding, potentially leading to misinterpretations. For comparison, polymeric ubiquitin variants with the same mass but different sites of cross-linking migrate very differently by SDS-PAGE [30], [31]. In addition, electron microscopy (EM) analysis of MUC2 [6], [9] may have been subject to artifacts or misinterpretation. Some three-way junctions were observed in atomic force microscopy (AFM) analysis of mucins isolated from pig small intestine [3], but as the mucin domain at the branch point and the mode of association were not established in that study, the junctions may correspond to isopeptide links or other mechanisms of lateral association of mucin polymers. It is clear that higher-order assembly of pre-formed amino-terminal region oligomers occurs under certain conditions through non-covalent association [9], and a dimeric structure of the VWD3 region may be incorporated into larger assemblies in this manner. Notably, a comparison of the MUC2D3 crystal structure with a low-resolution model, derived from EM, for such a higher-order assembly [20] is informative. A volume related by 2-fold symmetry in the EM reconstruction of a similar fragment may correspond to the MUC2D3 dimer structure presented here, whereas the 3-fold symmetry axis may be formed by other regions of the protein under certain conditions (Supplemental Fig. 9).

**Fig. 7:**
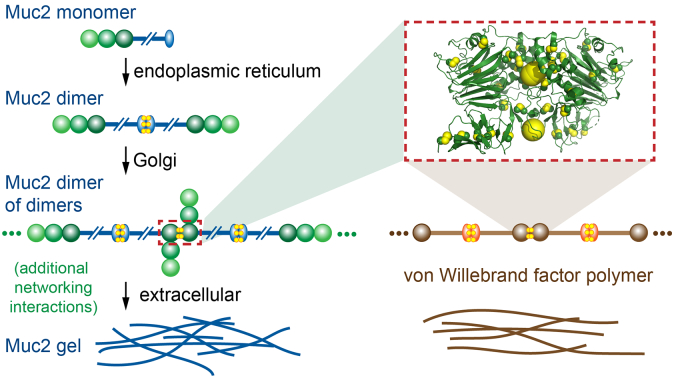
Schematic of MUC2 assembly. MUC2 D domains are shown as green spheres. The MUC2 cystine knot domain is shown as a blue oval. The MUC2D3 structure provides a homology model for dimerization of the paralogous domains of VWF. MUC2 may form additional lateral non-covalent and covalent interactions that contribute to network formation and protection of the intestinal epithelium.

In addition to adding a new perspective on mucin assembly, the MUC2D3 structure has implications for VWF polymerization (Fig. 7). Various VWF domains have been studied structurally, but the manner in which the amino terminal region forms covalent multimers has been elusive. An earlier study involving production of recombinant VWF amino-terminal fragments reported mixtures of monomers and dimers [32]. The disulfide connectivity of the monomeric population was analyzed under the assumption that the unpaired cysteines in that population are the same cysteines that engage with their partners across the dimer interface. However, an alternative interpretation is that the monomer population failed to dimerize because it was locally misfolded and exposed the wrong unpaired cysteine. As noted in the analysis of the VWF monomeric D3 structure [22], a conformational change would be required for intersubunit disulfide bonding of the presumed VWF interface cysteine, since its position within the final helix of the C8–3 domain is sterically incompatible with disulfide bonding across the dimer interface.

While it seems unlikely that the counterpart of MUC2 C1088 is found in VWF but not used for dimerization, the comparison of MUC2 and VWF offers some surprising insights into molecular evolution. At another position, an apparently conserved amino acid plays different roles in the two structures. K918, a conserved, surface-exposed lysine in MUC2, corresponds to K923 buried in the core of the β-sandwich in VWF (Supplementary Fig. 10). Although amino acid sequence homology is a powerful predictor of structure conservation [33], residues that are apparently aligned in primary structures occasionally appear in functionally different tertiary structural contexts [34]. Another example of this phenomenon is the change in connectivity of a conserved cysteine, as described above for MUC2 C1137.

In addition to the unexpected multimerization state of the MUC2 amino-terminal region, another important finding from the MUC2 structure is the nature of the interaction interface, which sheds light on assembly steps that happen downstream of the ER in the secretory pathway. The contacts between the MUC2D3 protomers involve conserved residues and are thus likely to be physiological, but the interface has unusual properties. The size of the interface is smaller than is typical, the interface is not contiguous but rather involves three distinct interacting regions, and many of the contacts are made by charged and polar residues. These observations are consistent with the stepwise assembly process of mucin multimers, in which carboxy-terminal dimerization [7] occurs early in the secretory pathway, and subsequent multimerization and additional cross-linking happen later in the pathway [6]. The prevalence of charged residues, including aspartates, glutamates, and histidines, near the interface (Fig. 4c, d) may be the key to controlling the site and timing of mucin and von Willebrand factor multimerization.

## Materials and Methods

### Protein production

The region spanning residues 858 to 1259 of human mucin 2 was expressed in the pcDNA3.1 plasmid downstream of the signal peptide from the protein QSOX1: MRRCNSGSGPPPSLLLLLLWLLAVPGANAAP. Cleavage is predicted to occur between the two adjacent alanines, leaving the amino acids alanine and proline fused to the amino terminus of the MUC2D3 domain. In addition, a His_6_ tag was fused to the carboxy terminus. The resulting plasmid was transiently transfected into HEK293F cells (ThermoFisher). Cells were maintained in FreeStyle 293 medium and transfected using the PEI Max reagent (Polysciences Inc.) with a 1:3 ratio (w/w) of DNA to PEI at a concentration of 1 million cells per milliliter. For protein preparations that yielded well-diffracting crystals, kifunensine was added at the time of transfection to a concentration of 5 μM to obtain protein containing high-mannose glycans. Five days after transfection, cells were removed from the cultures by centrifugation for 10 min at 500*g*. The culture medium was then further clarified by centrifugation for 30 min at 9500*g* and filtration through a 0.45-μm pore-size membrane. The MUC2D3 domain was purified from the medium by nickel-nitrilotriacetic acid chromatography followed by size exclusion chromatography in 25 mM Hepes buffer (pH 7.4), 250 mM NaCl, and 10% glycerol for crystallization, or 25 mM Hepes (pH 7.4) and 250 mM NaCl for other studies. The protein was concentrated to 5 mg/ml in a volume of about 500 μl, and 500 units of EndoH (about 550 ng) were added. A few hours later, the cleaved protein was used directly to prepare crystal trays. After cleavage by EndoH, the MUC2D3 preparation was stable at room temperature for weeks and continued to yield crystals, but the protein stock precipitated if stored in the cold.

The longer MUC2 fragments, spanning amino acids 21 to 1259 (D1D2D3) and 21 to 1397 (D1D2D3cysD1), were produced and purified essentially as for MUC2D3 except that kifunensine was not added and preparative gel filtration was done in 10 mM Hepes (pH 7.5) and 20 mM NaCl.

### Protein size determination using SEC-MALS

SEC-MALS was performed at a flow rate of 0.5 ml/min in a running buffer of 10 mM Tris (pH 7.4), 20 mM NaCl, and 0.02% sodium azide for the MUC2 fragments spanning residues 21–1259 (D1D2D3) and 21–1397 (D1D2D3CysD1), and in 25 mM Hepes (pH 7.4), 250 mM NaCl, and 0.02% sodium azide for MUC2D3. MUC2 D1D2D3 and D1D2D3CysD1 were analyzed on a Superose 6 10/300 column, and MUC2D3 was analyzed on a Superdex 200 Increase 10/300 column. Data were collected using a Dawn Heleos-II and an Optilab Trex (Wyatt Technology). Extinction coefficients were calculated using ExPASy ProtParam and were 1.419, 1.220, and 1.247 ml mg^− 1^ cm^− 1^ for the fragments MUC2D3, D1D2D3, and D1D2D3CysD1, respectively. Data recording and processing were performed using the ASTRA software (Wyatt Technology).

### Crystallization and structure determination

MUC2D3 possessing native glycans was crystallized by the hanging drop vapor diffusion method over a well solution containing 140 to 180 mM ammonium sulfate, 15% glycerol, 18% to 22% polyethylene glycol (PEG) 8000, and 5% PEG 400. Crystals of EndoH-treated MUC2D3 produced from cells treated with kifunensine were grown over a well solution containing 105 mM lithium sulfate, 100 mM citrate/phosphate buffer (pH 4.2), 10% glycerol, and 14% PEG 1500. Crystals that were washed in well solution and subjected to gel electrophoresis confirmed that the crystallized protein migrated identically to the protein stock solution and was therefore not a minor fraction with a different oligomerization state. Prior to flash freezing for data collection, crystals were transferred to a solution containing the same components but with the glycerol concentration increased to 25%. Data were collected on ESRF beamline ID23-2 and on the Swiss Light Source beamline PX-III.

The MUC2D3 structure was solved by molecular replacement (MR) using Phaser [35]. The search model was the structure of the monomeric VWF D3 domain (PDB ID: 6N29) spanning residues 865 to 1227. A single dominant MR solution was obtained. The MUC2D3 structure was iteratively refined and rebuilt on the basis of the MR solution using Phenix [36] and Coot [37], respectively, to produce the final model, which was verified by inspection of a combined simulated annealing omit map. Regions of the protein that were not visible or were uninterpretable in the omit map electron density contoured at 1 σ were not included in the model. Although some electron density was seen corresponding to additional monosaccharide modifications, carbohydrates were not included in the model if their orientations were not clear. Model geometry was evaluated using Molprobity [38], and the MUC2D3 structure model scored at the 80th percentile of structures in a similar resolution range.

### Sequence conservation analysis

The region of the MUC2D3 chain A structure spanning amino acids 858 to 1192 was used as the structure template for mapping the conservation scores obtained using Consurf [25]. The multiple sequence alignment (MSA) supplied for the Consurf analysis was generated from the corresponding region of manually collated mucin 2 sequences. A broad representation of intestinal mucins was chosen while avoiding the inclusion of sequences that are not true orthologs. Mucin 2 orthologs chosen were from the following: *Homo sapiens*, *Phascolarctos cinereus*, *Ailuropoda melanoleuca*, *Mus musculus*, *Sus scrofa*, *Rattus norvegicus*, *Cavia porcellus*, *Erinaceus europaeus*, *Oryctolagus cuniculus*, *Sorex araneus*, *Fukomys damarensis*, *Manis javanica*, *Meriones unguiculatus*, *Condylura cristata*, *Loxodonta Africana*, *Trichechus manatus latirostris*, *Pteropus alecto*, *Delphinapterus leucas*, *Bos taurus*, *Ovis aries*, *Lagenorhynchus obliquidens*, *Felis catus*, *Ursus arctos horribilis*, *Canis lupus*, *Equus caballus*, *Camelus dromedarius*, *Vombatus ursinus*, *Gallus gallus*, *Buceros rhinoceros silvestris*, *Aquila chrysaetos canadensis*, *Zonotrichia albicollis*, *Lonchura striata domestica*, *Dromaius novaehollandiae*, *Erythrura gouldiae*, *Crocodylus porosus*, *Tyto alba*, *Merops nubicus*, *Anolis carolinensis*, *Notechis scutatus*, *Pogona vitticeps*, *Python bivittatus*, *Ophiophagus hanna*, *Terrapene mexicana triunguis*, *Chelonia mydas*, *Xenopus laevis*, *Gekko japonicas*, *Danio rerio*, *Takifugu rubripes*, *Salmo salar*, *Esox lucius*, *Anabas testudineus*, *Seriola dumerili*, *Rhincodon typus*, *Astyanax mexicanus*, and *Latimeria chalumnae.* A comparable procedure was used for analysis of respiratory tract mucins and VWF.

### Accession numbers

The coordinates of the MUC2D3 structure have been deposited in the PDB with accession code 6RBF.

## Appendix A Supplementary data

Supplementary material Image 1
