# Supplementary material for: Intestinal Gel-Forming Mucins Polymerize by Disulfide-Mediated Dimerization of D3 Domains
Source: J Mol Biol. 2019 Sep 6;431(19):3740–52. doi: 10.1016/j.jmb.2019.07.018 (PMC6739602; doi:10.1016/j.jmb.2019.07.018)
Supplement: Supplementary file 1 — Supplementary material [file mmc1.docx]

**Supplemental Material**

**Supplemental Table**

Supplemental Table 1 Crystallographic data and refinement statistics

| **Data collection** | |
| --- | --- |
| Space group | *P*2_1_2_1_2 |
| Cell dimensions |  |
| a, b, c (Å) | 152.461, 156.925, 93.584 |
| α, β, γ (°) | 90, 90, 90 |
| Asymmetric unit | 2 dimers |
| Resolution (Å) | 48.35 - 2.70 (2.801 - 2.70) |
| Measured reflections | 840,121 (81,660) |
| Unique reflections | 62,040 (6087) |
| Completeness (%) | 99.85 (98.93) |
| Redundancy | 13.5 (13.4) |
| < I/σI > | 9.92 (1.30) |
| R_meas_ | 0.3245 (2.242) |
| R_pim_ | 0.08746 (0.6062) |
|  | |
| **Refinement** | |
| Resolution (Å) | 48.35 - 2.70 |
| Reflections in working set | 62,027 (6087) |
| Reflections in test set | 3100 (295) |
| R_work_/R_free_ | 0.217/0.283 |
| Number of protein atoms | 10,762 |
| Number of water molecules | 157 |
| Mean B-factor | 55.6 |
| Root mean square deviations |  |
| Bond length (Å) | 0.015 |
| Bond angle (°) | 1.59 |
| Ramachandran plot |  |
| Favored regions (%) | 89.7 |
| Additional allowed regions (%) | 9.4 |
| Disallowed regions (%) | 0.93 |

Values in parentheses are for highest resolution bins.

Chain A has no Ramachandran outliers.

**Supplemental Figures**

Supplemental Fig. 1


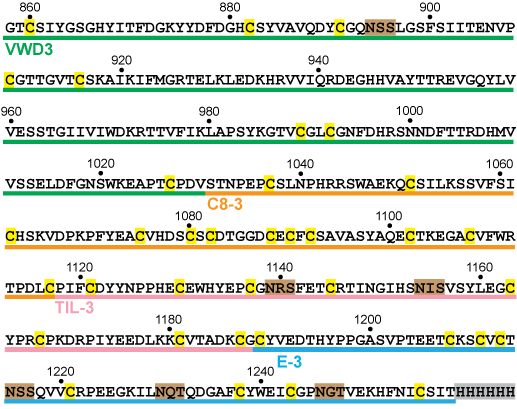


Supplemental Figure 1

Amino acid sequence of MUC2D3. Numbering is according to the MUC2 precursor (Uniprot Q02817). Domains are labeled and indicated by colored underlines, corresponding to Figure 2A. Cysteine residues are highlighted in yellow, consensus sites for N-linked glycosylation are shaded in brown, and the carboxy-terminal His_6_ tag added to aid in protein purification is indicated in gray.

Supplemental Fig. 2


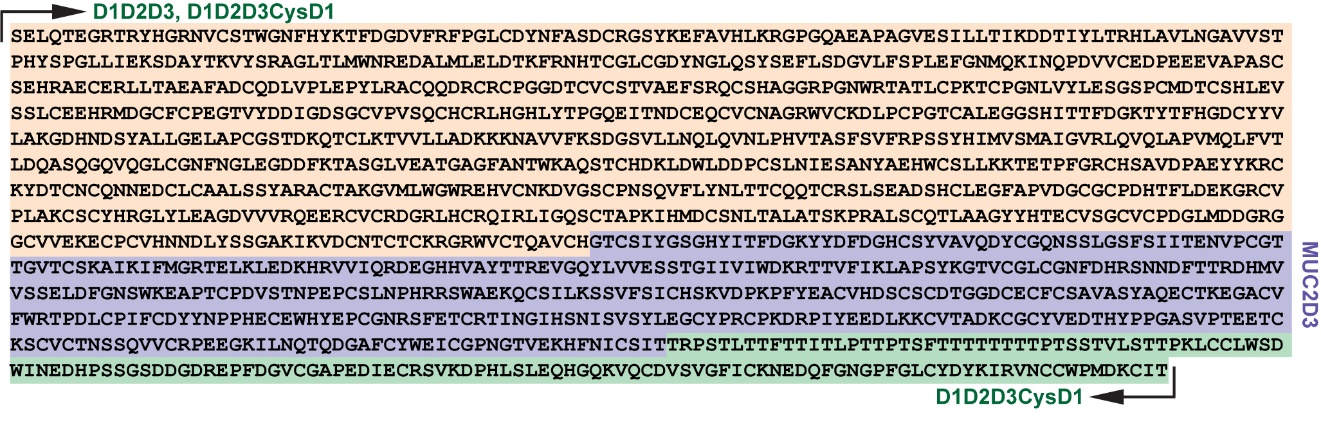


Supplemental Figure 2

Amino acid sequence of MUC2 constructs used for size determination. The MUC2D3 region is highlighted in violet. The D1D2D3 construct includes the regions highlighted in peach and violet, while the D1D2D3CysD1 spans the entire sequence shown.

Supplemental Fig. 3


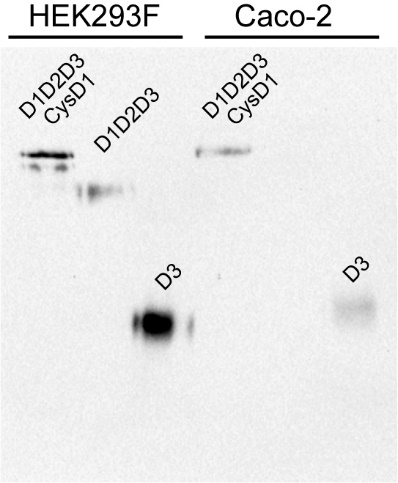


Supplemental Figure 3

Plasmids for three mucin amino-terminal fragments were transfected into Caco-2 cells. Culture media were collected after three days and dialyzed overnight against 25 mM sodium phosphate buffer, pH 7.5, 250 mM NaCl, and 10 mM imidazole. His_6_-tagged proteins were concentrated by binding to nickel-nitrilotriacetic acid beads. Eluates were applied to a 7.5% gel, along with purified proteins from HEK293F cell expression. His_6_-tagged proteins were identified by western using an antibody to the tag. The D1D2D3 fragment was not detected, but the other two fragments migrated similarly when produced from Caco-2 cells as from HEK293F cells.

Supplemental Fig. 4


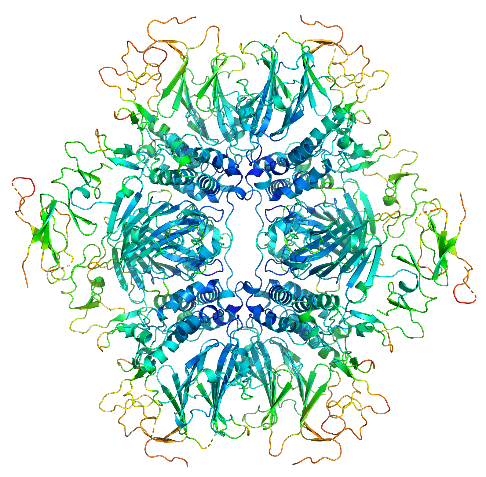


Supplemental Figure 4

The two MUC2D3 dimers in the crystal asymmetric unit form a compact octamer *via* crystallographic symmetry. The cartoon ribbon is colored according to B factor as in Figure 1c. The solvent-accessible surface area buried between the two dimers in the crystal asymmetric unit is only about 470 Å^2^. The solvent-accessible surface area buried between VWD3 domains (the β-sheet-rich domains along the central horizontal axis of the assembly) due to crystallographic symmetry is about 920 Å^2^.

Supplemental Fig. 5


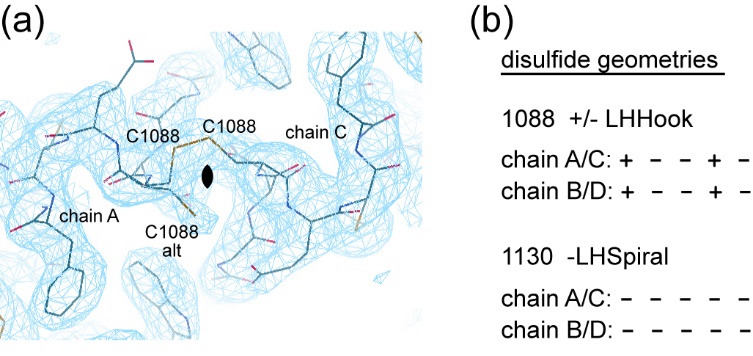


Supplemental Figure 5

The C1088-C1088 intersubunit disulfide is conformationally asymmetric. (a) The two-fold rotation axis that relates the two MUC2D3 dimers is perpendicular to the image, intersecting at the black symbol in the center. The C1088-C1088 disulfide does not obey this symmetry relation. The X1 dihedral angles of the two C1088 side chains are 76° and -69°. A minor population of C1088 in chain A exhibits an alternate rotamer (C1088 alt) incompatible with the disulfide bond. This population may have pre-existed in the crystals or been induced by synchrotron radiation. (b) The geometries of the two intersubunit disulfides are indicated according to Schmidt *et al*. (B. Schmidt, L. Ho, P.J. Hogg, Allosteric disulfide bonds. *Biochem* 45 (2006) 7429-7433.) The plus and minus symbols refer to the sign of the dihedral angles Χ1, Χ2, Χ3, Χ2’, Χ1’.

Supplemental Fig. 6

**D3 vs. D1**

859 TCSIYGSGHYITFDGKYYDFDGHCSYVAVQDYCGQNSSLGSFSIITENVPCGTTGVTCSK 918

36 VCSTWGNFHYKTFDGDVFRFPGLCDYNFASDCRG---SYKEFAVHLKRGPGQAEAPAGVE 92

919 AIKIFMGRTELKLEDKHRVVIQ----RDEGHHVAYTTREVGQYLVVESSTGIIVIWDKRT 974

93 SILLTIKDDTIYLT-RHLAVLNGAVVSTPHYSPGLLIEKSDAYTKVYSRAGLTLMWNRED 151

975 TVFIKLAPSYKGTVCGLCGNFDH-RSNNDFTTRDHMVVSSELDFGNSWK-EAP--TCPDV 1030

152 ALMLELDTKFRNHTCGLCGDYNGLQSYSEFLSDG--VLFSPLEFGNMQKINQPDVVCEDP 209

1031 STNPEPCSLNPHRRSWAEKQCSILKSSVFSICHSKVDPKPFYEACVHDSCSCDTGGDCEC 1090

210 EEEVAPASCSEHR---AECE-RLLTAEAFADCQDLVPLEPYLRACQQDRCRC-PGGD-TC 263

1091 FCSAVASYAQECTKEGACVF-WRTPDLCPIFCDYYNPPHECEWHYEPCGNRSFETCRTIN 1149

264 VCSTVAEFSRQCSHAGGRPGNWRTATLCPKTCPG-------NLVYLESGSPCMDTCSHLE 316

1150 GIHSNISVSYLEGCYPRCPKDRPIYEEDLKKCVTADKCGCYVEDTHYPPGASVPTEETCK 1209

317 -VSSLCEEHRMDGCF--CPEGTVYDDIGDSGCVPVSQCHCRLHGHLYTPGQEITND--CE 371

1210 SCVCTNSSQVVCR 1222

372 QCVC-NAGRWVCK 383

**D3 vs. D2**

858 GTCSIYGSGHYITFDGKYYDFDGHCSYVAVQDYCGQNSSLGSFSIITENVPCGTTG-VTC 916

389 GTCALEGGSHITTFDGKTYTFHGDCYYVLAK---GDHND--SYALLGELAPCGSTDKQTC 443

917 SKAIKIFMGRTE--LKLEDKHRVVIQRDEGH--HVAYTT---REVGQYLVVESSTGI--I 967

444 LKTVVLLADKKKNAVVFKSDGSVLLNQLQVNLPHVTASFSVFRPSSYHIMVSMAIGVRLQ 503

968 VIWDKRTTVFIKLAPSYKGTVCGLCGNFDHRSNNDFTTRDHMVVSSELDFGNSWKEAPTC 1027

504 VQLAPVMQLFVTLDQASQGQVQGLCGNFNGLEGDDFKTASGLVEATGAGFANTWKAQSTC 563

1028 PD-VSTNPEPCSLNPHRRSWAEKQCSILKSSV--FSICHSKVDPKPFYEACVHDSCSCDT 1084

564 HDKLDWLDDPCSLNIESANYAEHWCSLLKKTETPFGRCHSAVDPAEYYKRCKYDTCNCQN 623

1085 GGDCECFCSAVASYAQECTKEGACVF-WRTPDLCPIFCDYYNPPHECEWHYEPCGNRSF- 1142

624 NED--CLCAALSSYARACTAKGVMLWGWRE--------------HVCNKDVGSCPNSQVF 667

1143 --------ETCRTINGIHSNISVSYLEGCYP----RCPKDRPIYEEDLKKCVTADKCGCY 1190

668 LYNLTTCQQTCRSLSEADSHC----LEGFAPVDGCGCPDH--TFLDEKGRCVPLAKCSCY 721

1191 VEDTHYPPGASVPTEETCKSCVCTNSSQVVCR 1222

722 HRGLYLEAGDVVVRQE--ERCVCRD-GRLHCR 750

Supplemental Figure 6

Alignment of MUC2D3 (top sequences) with the MUC2 D1 and D2 domains. Amino acid numbers are given at the ends of each row. Cysteines participating in intermolecular disulfides are highlighted in red. Conserved cysteines are highlighted in yellow. Cysteines that do not appear in the identical positions or lack partners in the alignment are highlighted in green.

Supplemental Fig. 7


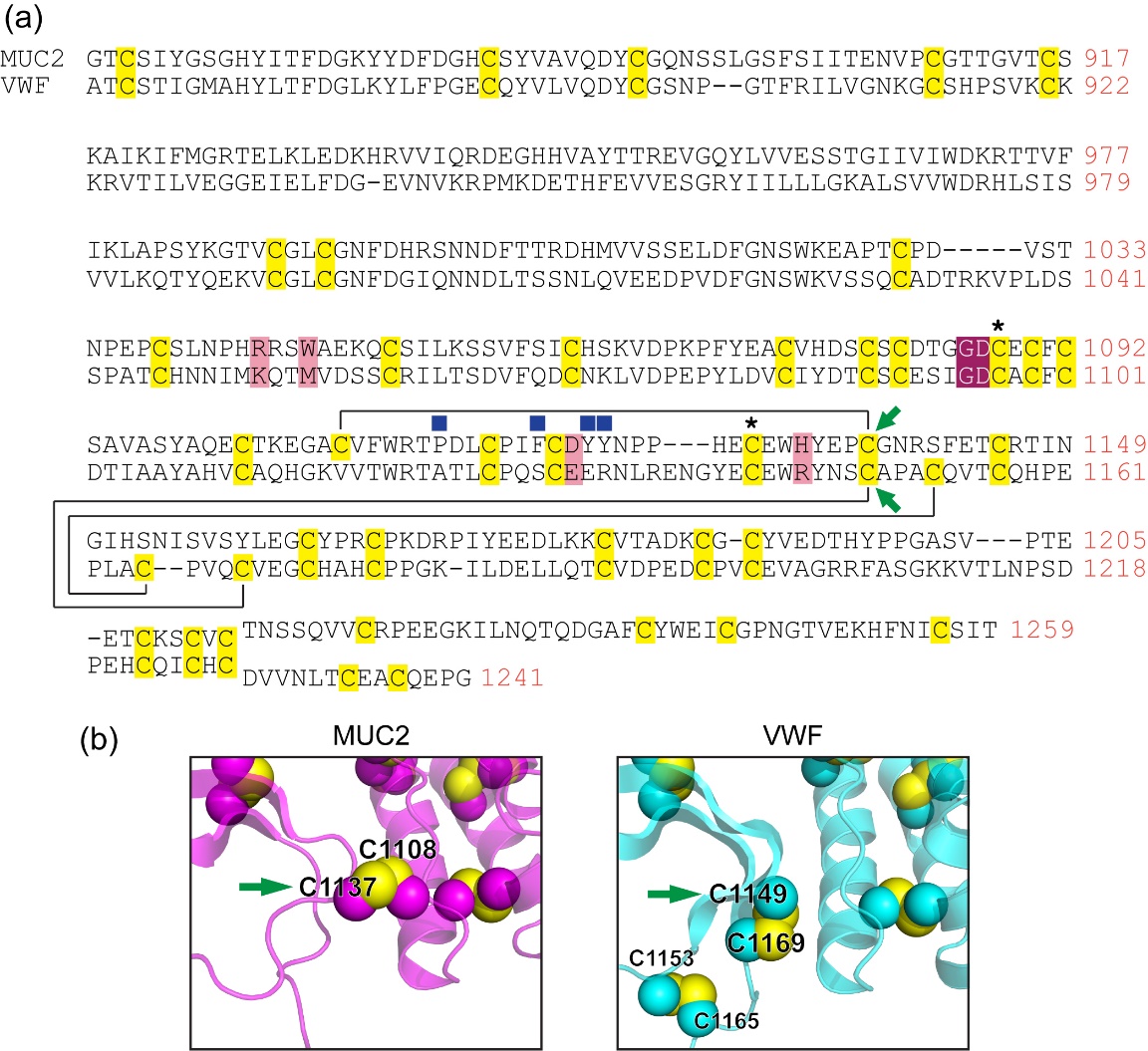


Supplemental Figure 7

Comparison of MUC2 and VWF. (a) Sequence alignment in region of MUC2D3. Amino acids at the end of each line are numbered according to Uniprot (MUC2: Q02817 and VWF: L8E853). The carboxy termini of the MUC2 and VWF sequences are not juxtaposed because a meaningful alignment cannot be made for this region. MUC2 lacks one of the VWF disulfides and shows a different connectivity for another, as indicated by the black lines connecting the cysteines (green arrows) involved in these disulfides. Asterisks indicate the cysteines making intersubunit disulfides. Blue squares indicate the MUC2 amino acids contributing to the hydrophobic interaction shown in Figure 4e. These positions are not conserved in VWF, but homology modeling of the VWF dimer (not shown) suggests that the polar residues that replace some of the aromatics may make hydrogen bonds to the backbone of the opposite subunit in VWF. Other interface amino acids are identical (magenta) or similar (pink) between MUC2 and VWF. (b) Change of disulfide connectivity in MUC2 vs. VWF. The green arrows indicate the cysteines that are conserved in the sequence alignment (MUC2 C1137 and VWF C1149). MUC2 C1137 is bonded to a cysteine just following the final helix in the C8-3 region, whereas VWF C1149 is bonded to a cysteine farther along in the TIL-3 domain. The extra disulfide in VWF, linking C1153 to C1165, is also evident.

Supplemental Fig. 8


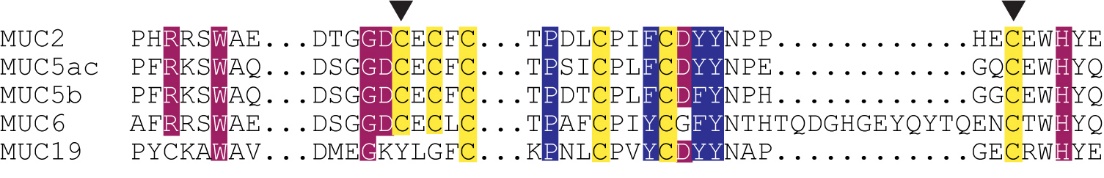


Supplemental Figure 8

Alignment of human gel-forming mucin sequences in the regions corresponding to the MUC2 dimer interface. Cysteines involved in intersubunit disulfide bonds in the MUC2D3 structure are indicated by arrowheads. Amino acids highlighted in magenta correspond to those making polar interactions across the dimer interface. Amino acids highlighted in blue correspond to those participating in intermolecular hydrophobic interactions.

Supplemental Fig. 9


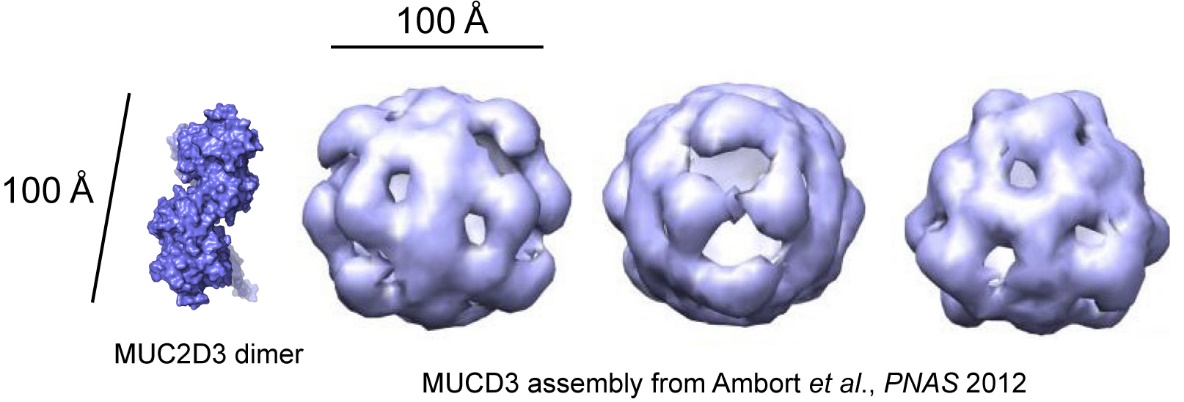


Supplemental Figure 9

A comparison of the MUC2D3 dimer structure with low-resolution EM reconstructions previously reported for a VWD3-containing region of MUC2 fused to green fluorescent protein [20]. A molecular surface representation of the crystallographic dimer structure is shown on the left, scaled to the volume representations of the EM structure.

Supplemental Fig. 10


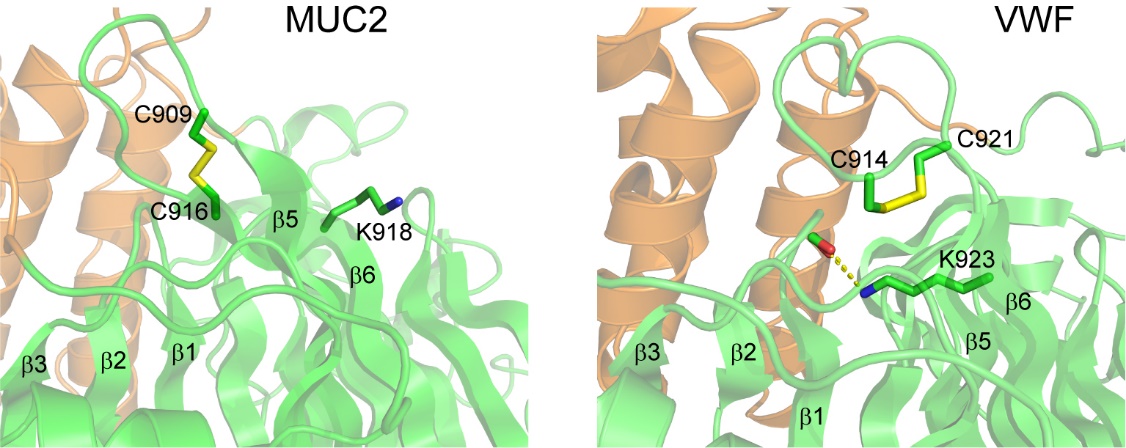


Supplemental Figure 10

A lysine conserved according to sequence alignment is in a structurally different environment in MUC2 compared to VWF. The side chain of MUC2 K918 is solvent-exposed in the MUC2D3 structure, whereas the comparable lysine in VWF, K923, points toward the center of the VWD3 domain β-sheet. The buried VWF lysine is in position to hydrogen bond with two backbone carbonyls on the loop between β-strands β1 and β2.
